# Association of MiR-27a-5p with chronic obstructive pulmonary disease and its role in human bronchial epithelial cell injury: A case-control and in vitro experimental study

**DOI:** 10.18332/tid/219984

**Published:** 2026-05-26

**Authors:** Jingfan Wang, Ping Zhang, Yonggang Qin

**Affiliations:** 1 Department of Respiratory and Critical Care Medicine, Hanyang Hospital Affiliated to Wuhan University of Science and Technology, Wuhan, China

**Keywords:** chronic obstructive pulmonary disease, miR-27a-5p, ABL2

## Abstract

**INTRODUCTION:**

Chronic obstructive pulmonary disease (COPD) lacks effective early diagnostic biomarkers, and the role of miR-27a-5p in COPD pathogenesis remains unclear. This study aimed to explore its inflammatory regulatory mechanism.

**METHODS:**

We enrolled COPD patients and healthy controls, measuring miR-27a-5p and inflammatory cytokines (TNF-α, IL-6, IL-1β) in serum and bronchoalveolar lavage fluid. Multivariate logistic regression and ROC curves were used to analyze disease association and diagnostic efficacy. A cigarette smoke extract-induced bronchial epithelial cell model was used with miRNA transfection and dual-luciferase assays to validate the target gene and mechanism.

**RESULTS:**

MiR-27a-5p was significantly upregulated in COPD patients (p<0.001), and its expression was associated with COPD alongside smoking status and pulmonary function (adjusted odds ratio, AOR=22.53; 95% CI: 12.31–41.30, p<0.001). It showed strong diagnostic efficacy (AUC=0.96; 95% CI: 0.92–0.99). In vitro, miR-27a-5p overexpression exacerbated inflammation, while inhibition alleviated it. Mechanistically, miR-27a-5p directly targeted ABL2 to regulate inflammatory cytokine release.

**CONCLUSIONS:**

MiR-27a-5p within our study was identified as a promising biomarker for COPD susceptibility and modulated airway inflammation via the miR-27a-5p-ABL2 axis.

## INTRODUCTION

Chronic obstructive pulmonary disease (COPD) encompasses chronic bronchitis and emphysema, resulting from long-term exposure to noxious gases and particles^1^. Smoking is a primary etiological factor^2^. Its key features include airway remodeling and alveolar destruction, which collectively cause persistent airflow limitation^3^. Approximately 3 million people die from COPD annually, yet current management remains largely reliant on bronchodilators, triple inhalation therapy, and α-1 antitrypsin augmentation therapy^4,5^. Effective clinical therapies for COPD remain to be developed^6^. Excessive expression of inflammatory factors and activation of inflammatory signaling pathways play critical roles in COPD pathogenesis^7^. As the first line of defense, bronchial epithelial cells secrete mediators that can trigger immune and inflammatory responses^8,9^. Thus, exploring the molecular mechanisms in bronchial epithelial cells may facilitate the development of adjunctive therapies to improve clinical management of COPD patients.

MicroRNAs (miRNAs) play key roles in various physiological and pathological processes, including cell proliferation and inflammatory responses^10,11^. As a well-studied member of this family, miR-27a-5p is associated with multiple diseases due to its abnormal expression. In type 2 diabetes, miR-27a-5p induces impaired glucose tolerance by inhibiting insulin secretion from pancreatic β cells^12^. The association between miR-27a-5p and COPD has attracted considerable attention. Studies have demonstrated that among miRNAs in lung fibroblasts from COPD patients, miR-27a-5p is the most significantly upregulated under transforming growth factor-β (TGF-β) regulation^13^. Given that TGF-β plays a critical role in COPD airway remodeling, its induction of miR-27a-5p overexpression may contribute to abnormal tissue repair and remodeling in COPD by regulating target gene transcription. Thus, miR-27a-5p may represent a key target for elucidating COPD pathogenesis and developing therapies.

ABL2, a target of miR-27a-5p, is a tyrosine kinase that participates in tissue remodeling by regulating inflammatory pathways (e.g. NF-κB and MAPK) and apoptosis – processes closely aligned with the core pathological features of COPD. Thus, investigating the miR-27a-5p/ABL2 axis may help elucidate the role of miR-27a-5p in COPD pathogenesis. This study provides novel insights into the association between miR-27a-5p and COPD, as well as its underlying biological mechanism, laying a preliminary theoretical foundation for further research into COPD-related regulatory pathways.

## METHODS

### Study design and population

This study adopted a case-control design combined with a subsequent *in vitro* experimental research arm. For the clinical component: between January 2024 and December 2024, 74 patients diagnosed with COPD (meeting the 2021 Revised Edition of Guidelines for the Diagnosis and Treatment of Chronic Obstructive Pulmonary Disease)^14^ were recruited from the Department of Respiratory Medicine at Hanyang Hospital affiliated to Wuhan University of Science and Technology (COPD group). Consecutive sampling was used. Meanwhile, 63 healthy individuals from the hospital’s Health Management Center who underwent physical check-ups during the same period were enrolled as the control group (exclusion criteria: pulmonary disorders, autoimmune conditions, recent infection histories). No matching was performed. All participants provided informed consent, and the study was approved by the Ethics Committee of Hanyang Hospital Affiliated to Wuhan University of Science and Technology (Approval number: HYLL-2023-035).

### Cell cultures and reagents

Human bronchial epithelial cells (HBECs) were purchased from the Chinese Academy of Sciences Cell Bank and cultured in DMEM medium (HyClone) containing 10% fetal bovine serum (Gibco), 100 U/mL penicillin, and 100 μg/mL streptomycin. The cells were passaged to the 3rd to 5th generation for experimental use. After culturing PBECs in the logarithmic growth phase for 48 hours, the cells were collected for the detection of miR-27a-5p expression changes.

### Constructing viral vectors

When constructing ABL2 overexpression and silencing HBECs cell lines, the cells were cultured in Lonza-specific medium. The vectors and reagents used included pcDNA3.1-ABL2, empty vector pcDNA3.1, ABL2-siRNA, negative control siRNA, Lipofectamine 3000, G418, puromycin, and ABL2 antibody; For the construction of overexpression cell lines, log-phase PBEC cells were seeded in triplicate into 6-well plates, transfected according to the reagent instructions, and then selected with medium containing 800 μg/mL G418 for 2 weeks, followed by expansion of single clones; silencing cell line construction involved seeding PBEC in three groups, transfecting at a final concentration of 50 nM, and screening for stable silencing cell lines using 2 μg/mL puromycin.

### Sample plasma and synovial fluid collection

All study participants had 5 mL of peripheral venous blood collected in the morning on an empty stomach. The blood was centrifuged at 3000 rpm for 10 minutes, the serum was separated, and stored at -80°C for future use. Bronchoalveolar lavage fluid (BALF) was collected from COPD patients and the control group. During fiberoptic bronchoscopy, 20 mL of sterile saline was injected into the middle lobe or lingular lobe, and the lavage fluid was recovered. It was then centrifuged at 4°C and 1500 rpm for 10 minutes, and the supernatant was collected and stored at -80°C.

### Cell transfection

We set up four groups: a blank control (untransfected), a negative control (transfected with miR-NC), a miR-27a-5p mimic group (transfected with miR-27a-5p mimic), and a miR-27a-5p inhibitor group (transfected with miR-27a-5p inhibitor). Transfection was performed with Lipofectamine 3000 (Invitrogen). PBECs were placed into 6-well plates (5×10^5^ cells per well) one day prior to transfection. Upon reaching 70–80% confluence, miRNA mimics, inhibitors, or negative controls (final concentration 50 nM) were blended with the transfection reagent according to the manufacturer’s instructions, added to the cell culture medium, and incubated for 48 hours before being utilized in later experiments.

### RNA extraction and real‑time PCR

Total RNA was extracted from cells and serum using the Total RNA Extraction Kit (DP419). The obtained RNA was reverse transcribed into cDNA using the High-Capacity cDNA Reverse Transcription Kit (CNY). The average threshold cycle was used to calculate relative expression using the Livak method with ACTB or GAPDH as internal controls for the TaqMan probes. After determining the concentration using a micro-nucleic acid quantifier, the RNA samples were stored at -80°C. The SYBR Green method was used to detect complementary deoxyribonucleic acid (cDNA).

### Dual‑luciferase reporter gene assay

The targeted binding of miR-27a-5p to ABL2 was validated via a dual-luciferase reporter assay. Constructed reporter vectors (obtained from GeneCopoeia) carrying the wild-type (WT) or mutant (MUT) 3’UTR of ABL2 were co-transfected into 293T cells along with the miR-27a-5p mimic or miR-NC. Forty-eight hours following transfection, the activities of firefly luciferase and sea squirt luciferase were determined using a Promega dual-luciferase detection kit, and relative luciferase activity was calculated as the ratio of firefly luciferase activity to sea squirt luciferase activity. Target gene prediction was performed using the TargetScan, miRDB, and miRTarBase databases.

### Detection of cellular inflammatory factors

To clarify the relationship between miR-27a-5p and inflammatory factors (TNF-α, IL-6, IL-1β), the following groups were set up in the experiment: control group (untreated); CSE treatment group; CSE + miR-27a-5p negative control (NC) group; CSE + miR-27a-5p overexpression group; and CSE + miR-27a-5p inhibition group. After 48 hours of treatment, the cell supernatants were collected, and the concentrations of TNF-α, IL-6, and IL-1β were detected using ELISA. The regulatory effect of miR-27a-5p on the secretion of inflammatory factors was analyzed; at the same time, the molecular pathways of its regulation were clarified through target gene verification experiments.

### Statistical analysis

Data analysis was performed using SPSS 26.0 software (IBM Corp., Armonk, NY, USA). Quantitative data are expressed as mean ± standard deviation (SD). Comparisons between two groups used the independent samples t-test, multiple groups used one-way ANOVA (pairwise comparisons via LSD-t test). To identify factors associated with current COPD status, multivariate logistic regression models (dependent variable: COPD status; covariates: miR-27a-5p expression, smoking status, pulmonary function parameters) were fitted to estimate odds ratios (ORs) and 95% confidence intervals (CIs). For diagnostic performance, receiver operating characteristic (ROC) curves were plotted to determine the area under the curve (AUC) and its 95% CI^15^. Single group comparisons were conducted with significance set at p<0.05 (no multiple comparison correction), and error bars represent standard errors.

## RESULTS

### Clinical baseline characteristics of COPD patients and non‑COPD individuals

**Figure 1:**
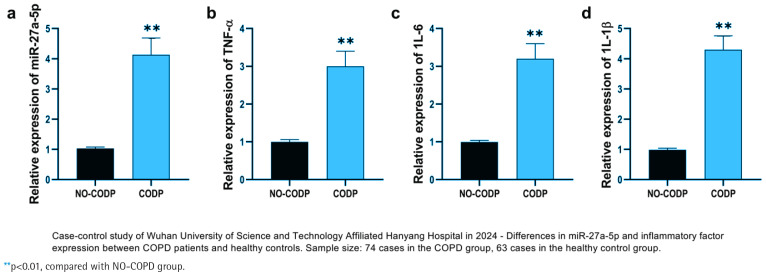
Relative expression levels of miR-27a-5p and inflammatory cytokines in NO-COPD and COPD samples: a) Comparison of miR-27a-5p levels between NO-COPD and COPD groups; b) Comparison of TNF-α mRNA levels between NO-COPD and COPD groups; c) Comparison of IL-6 mRNA levels between NO-COPD and COPD groups; and d) Comparison of IL-1β mRNA levels between NO-COPD and COPD groups. Black bars represent the NO-COPD group, gray bars represent the COPD group

This study included non-COPD individuals and COPD patients, analyzing their clinical baseline data. Results from lung function testing showed that COPD patients had a significantly lower forced expiratory volume in one second to forced vital capacity ratio (FEV_1_/FVC %) compared with the non-COPD group. In addition, the percentage of forced expiratory volume in one second relative to the predicted value (FEV_1_ %) was notably reduced in the COPD cohort (p*<*0.001) (Table 1). However, age, body mass index (BMI, kg/m^2^), neutrophil count (Neu, 10^9^/L), eosinophil count (Eos, 10^9^/L), basophil count (Bas, 10^9^/L), and lymphocyte count (Lym, 10^9^/L) showed no statistically significant differences. Compared with the healthy control group, the relative expression level of miR-27a-5p in the peripheral blood of COPD patients was significantly increased (p*<*0.001); simultaneously, the relative expression levels of inflammatory factors TNF-α, IL-6, and IL-1β also increased synchronously (all p*<*0.001) (Figure 1).

**Table 1:** Comparison of clinical baseline characteristics between COPD patients and non-COPD population

*Characteristics*	*Non-COPD Mean ± SD*	*COPD Mean ± SD*	*p*
Total, n	63	74	
Age (years)	52.7 ± 6.2	54.04 ± 7.52	0.1371
BMI (kg/m^2^)	26.16 ± 2.94	26.86 ± 3.86	0.1223
FEV_1_/FVC (%)	82.16 ± 7.7	53.25 ± 11.11	<0.001
FEV_1_ (%)	88.39 ± 5.61	65.95 ± 15.62	<0.001
Neu (10^9^/L)	5.98 ± 1.43	6.28 ± 1.78	0.1444
Eos (10^9^/L)	0.329 ± 0.32	0.34 ± 0.202	0.3762
Bas (10^9^/L)	0.031 ± 0.015	0.03 ± 0.016	0.4923
Lym (10^9^/L)	3.72 ± 1.96	3.68 ± 2.07	0.4583

**Table 2:** Multivariate logistic regression analysis of risk factors for COPD onset

*Variables*	*AOR*	*95% CI*	*p*
Age (years)	0.985	0.387–2.502	0.947
BMI (kg/m^2^)	0.862	0.348–2.133	0.748
Smoke	6.655	2.380–18.606	<0.001
FEV_1_/FVC (%)	3.034	1.192–7.722	0.020
FEV_1_ (%)	3.400	1.296–8.920	0.013
Neu (10^9^/L)	2.152	0.827–5.597	0.116
Eos (10^9^/L)	1.951	0.767–4.968	0.161
Bas (10^9^/L)	0.781	0.311–1.957	0.597
Lym (10^9^/L)	0.467	0.177–1.231	0.124
miR-27a-5p	22.534	7.388–68.732	<0.001

AOR: adjusted odds ratio.

### Changes in miR‑27a‑5p and inflammatory factor expression levels in COPD patients

The expression of miR-27a-5p and key inflammatory factor mRNAs was detected in NO-COPD individuals and COPD patients. The relative expression level of miR-27a-5p in COPD patient samples was significantly higher than in non-COPD individuals (p*<*0.01) (Figure 1a). miR-27a-5p exhibits a trend of high expression in COPD patients and may be involved in the onset and progression of the disease. Tumor necrosis factor-α (TNF-α) mRNA, interleukin-6 (IL-6) mRNA, and interleukin-1β (IL-1β) mRNA were also significantly up-regulated in COPD patients (p*<*0.01) (Figures 1b–1d).

### Multifactorial analysis of COPD incidence risk

Using multivariate logistic regression analysis, we investigated the influence of various factors associated with COPD. Smoking status was the strongest factor associated with COPD (AOR=6.66; 95% CI: 2.38–18.61, p*<*0.001). The AOR values for the lung function indicators FEV_1_/FVC (%) and FEV_1_ (%) were 3.03 (95% CI: 1.19–7.72, p=0.020) and 3.40 (95% CI: 1.30–8.92, p=0.013), respectively. Additionally, in the unadjusted analysis, miR-27a-5p was associated with COPD status (OR=22.53, 95% CI: 7.39–68.73, p*<*0.001).

### CSE alters the expression of miR-27a-5p and inflammatory factors in HBECs

**Figure 2:**
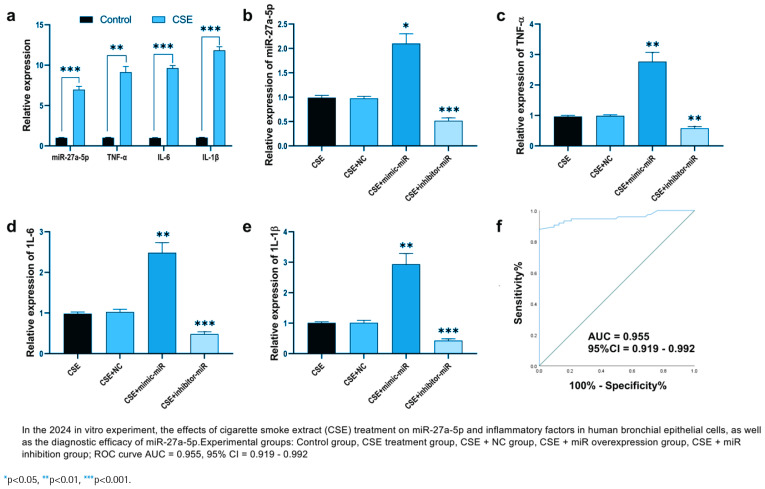
Functional analysis of miR-27a-5p in CSE treated PBECs and its diagnostic efficacy for COPD: a) Relative expression of miR-27a-5p, TNF-α, IL-6, and IL-1β in PBECs with or without CSE treatment; b) MiR-27a-5p expression in CSE-treated PBECs after transfection with miR-27a-5p mimic, inhibitor, or negative control; c) Relative expression of TNF-α in CSE-treated PBECs under different miR-27a-5p regulation conditions; d) Relative expression of IL-6 in CSE-treated PBECs under different miR-27a-5p regulation conditions; e) Relative expression of IL-1β in CSE-treated PBECs under different miR-27a-5p regulation conditions; and f) ROC curve analysis of miR-27a-5p for distinguishing COPD patients from controls

We investigated the effects of cigarette smoke extract (CSE) treatment on the expression of miR-27a-5p and inflammatory factors in HBECs. Following CSE exposure, the relative expression levels of miR-27a-5p and inflammatory factors in HBECs were significantly increased (p*<*0.01) (Figure 2a). Based on CSE treatment, HBECs were transfected with miR-27a-5p mimics (CSE + miR mimic), inhibitors (CSE + miR inhibitor), and negative controls (CSE + NC). Results indicated that after introducing miR mimics via transfection, the relative expression of miR-27a-5p was significantly greater than in the CSE + NC group (p<0.05) (Figure 2b). When miR inhibitors were transfected, the relative expression of miR-27a-5p was markedly lower than that in the CSE + NC group (p<0.001) (Figure 2b), confirming the successful development of cell models with miR-27a-5p overexpression and inhibition.

Further examination of how inflammatory factors’ expression alters revealed that their relative levels were notably increased in the CSE + miR mimic group and significantly decreased in the CSE + miR inhibitor group (p<0.01) (Figures 2c–2e). The diagnostic value of miR-27a-5p for COPD was evaluated using a ROC curve, with an AUC of 0.955 (95% CI: 0.919–0.992).

In the human bronchial epithelial cell model treated by CSE, the expression level of miR-27a-5p in the cells significantly increased, and the secretion levels of TNF-α, IL-6, and IL-1β also significantly increased (p<0.01 or p<0.001) (Figure 2a). Further verification through cell transfection experiments showed that overexpression of miR-27a-5p could significantly promote the release of TNF-α, IL-6, and IL-1β induced by CSE (p*<*0.01); while inhibiting the expression of miR-27a-5p could reverse the high expression of inflammatory factors caused by CSE (p*<*0.001) (Figures 2c–2e). Combined with the subsequent validation results of target genes (miR-27a-5p targets and inhibits ABL2, and ABL2 can regulate the NF-κB/MAPK inflammatory pathway, (Figures 2d and 2e), it suggests that miR-27a-5p activates the inflammatory signaling pathway by targeting ABL2, thereby promoting the secretion of TNF-α, IL-6, and IL-1β.

### MiR-27a-5p target gene prediction and functional analysis

Target gene prediction yielded 43 common target genes (Supplementary file Figure S1a). Pathway analysis of these target genes (Supplementary file Figure S1b) revealed that they were primarily enriched in the MAPK signaling pathway, Ras signaling pathway, and Hippo signaling pathway, suggesting that miR-27a-5p may participate in the pathological process of COPD by regulating these signaling pathways. GO functional analysis (Supplementary file Figure S1c) showed that the target genes were significantly enriched in biological processes such as metabolic processes, stress responses, and transcriptional regulation, as well as molecular functions and cellular components, providing direction for subsequent screening of key target genes.

MiR-27a-5p targets ABL2, a tyrosine kinase that participates in tissue remodeling by regulating inflammatory pathways such as NF-κB and MAPK, as well as apoptosis. This is closely consistent with the core pathological processes of COPD, and exploring their association may shed light on how miR-27a-5p acts in COPD. Sequence alignment identified complementary binding sites between miR-27a-5p and the ABL2 3’UTR (Supplementary file Figure S1d). Through a dual luciferase reporter gene assay, co-transfecting WT-miR-27a-5p with the ABL2 wild-type reporter vector (Control+CV-ABL2) led to a marked rise in relative luciferase activity (p*<*0.05). However, co-transfection with MUT-miR-27a-5p or ABL2 interference (Control+siRNA-ABL2) resulted in no significant changes in luciferase activity (p<0.001) (Supplementary file Figure S1e), confirming that miR-27a-5p can directly target and bind to ABL2, thereby regulating its expression.

### MiR-27a-5p regulates CSE-induced inflammatory response in HBECs by targeting ABL2

Compared with non-COPD individuals, the relative expression level of ABL2 was significantly higher in COPD patient samples. In CSE-treated PHBECs, ABL2 expression was also significantly elevated (p<0.01) (Supplementary file Figure S2a), suggesting that ABL2 may be involved in the inflammatory response in COPD. Following CSE treatment, overexpression of ABL2 upregulated miR-27a-5p expression (p<0.01) (Supplementary file Figure S2c), while inhibition of ABL2 downregulated miR-27a-5p (p<0.001), indicating a mutual regulatory relationship between miR-27a-5p and ABL2, with both jointly participating in the regulation of cellular functions.

Detection of TNF-α, IL-6, and IL-1β expression showed that ABL2 overexpression (CSE+CV-ABL2) significantly promoted CSE-induced expression of TNF-α (p<0.01) (Supplementary file Figure S2d), IL-6 (p<0.01) (Supplementary file Figure S2e), and IL-1β (p<0.01) (Supplementary file Figure S2f). In contrast, ABL2 knockdown (CSE+siRNA-ABL2) inhibited the expression of these inflammatory factors (p*<*0.001). Furthermore, compared with ABL2 overexpression alone, co-transfection of the ABL2 overexpression vector with the miR-27a-5p mimic (CSE+CV-ABL2+miR mimic) led to further upregulation of inflammatory factor expression (p*<*0.001). These findings confirm that miR-27a-5p exacerbates CSE-induced inflammatory responses in PHBECs by targeting ABL2, providing new molecular regulatory evidence for the mechanisms underlying airway inflammation in COPD.

## DISCUSION

This study explores the role of miR-27a-5p in chronic obstructive pulmonary disease (COPD) through clinical and cellular experiments. We found that miR-27a-5p is highly expressed in COPD patients and correlates with disease risk^16^; in cellular models, it regulates inflammatory responses induced by cigarette smoke extract (CSE), and mechanistically targets ABL2 to form a regulatory axis affecting inflammatory factor release. These results clarify a novel molecular pathway involved in COPD pathogenesis^17^.

Existing studies have identified multiple miRNAs (e.g. miR-126, miR-146a) as potential COPD biomarkers or regulatory factors, but their specific mechanisms in inflammatory processes remain incompletely defined^18^. Our study extends this field by confirming that miR-27a-5p acts as a pro-inflammatory regulator: unlike previous studies focusing solely on miRNA expression profiles, we link miR-27a-5p to a specific target (ABL2) and its downstream inflammatory pathways (NF-κB/MAPK), providing a more complete mechanistic chain. Additionally, the high diagnostic efficacy of miR-27a-5p complements the limitations of traditional inflammatory factor biomarkers, which often lack specificity in early COPD^19^.

### Strengths and limitations

A key strength of this study is the integration of clinical case-control data, cellular functional experiments, and bioinformatic analyses, which bridges the gap between clinical observations and molecular mechanisms. However, limitations exist: we did not perform *a priori* sample size calculation, which may reduce the power to detect small effect sizes; moreover, the study only validated *in vitro* mechanisms, lacking *in vivo* animal experiments to confirm the role of the miR-27a-5p/ABL2 axis in airway remodeling. These limitations should be addressed in future research by expanding sample sizes (via pre-calculation) and using animal models to verify any possible therapeutic potential^20^.

### Implications

The key implications of these findings include three aspects: First, with an AUC of 0.955 in ROC analysis, miR-27a-5p shows potential as a non-invasive biomarker for early COPD screening, which can assist in identifying high-risk populations (e.g. long-term smokers) and compensate for the limitations of traditional lung function tests in early diagnosis. Second, the miR-27a-5p/ABL2 regulatory axis provides additional information as a potential target for anti-inflammatory therapy in COPD, supporting the development of miR-27a-5p inhibitors or ABL2 agonists, results which, however, would need to be verified with additional clinical studies. Third, subsequent studies may validate the correlation between miR-27a-5p and COPD clinical indicators (e.g. FEV1 (%) predicted value, acute exacerbation frequency), enabling it to serve as a dynamic monitoring index for disease progression and individualized treatment adjustment.

## CONCLUSIONS

This study confirms that miR-27a-5p is highly expressed in COPD patients and regulates airway inflammation by targeting ABL2 to activate the NF-κB/MAPK pathway, thereby participating in COPD pathogenesis. Overall, this research enriches the understanding of COPD’s molecular mechanisms and offers new evidence for the precision diagnosis of COPD. Further clinical studies are needed to corroborate our findings.

## Supplementary Material



## Data Availability

Data sharing is not applicable to this article as no new data were created.
