# Supplementary material for: Association of MiR-27a-5p with chronic obstructive pulmonary disease and its role in human bronchial epithelial cell injury: A case-control and in vitro experimental study
Source: Tob Induc Dis. 2026 May 26;24:10.18332/tid/219984. doi: 10.18332/tid/219984 (PMC13205640; doi:10.18332/tid/219984)
Supplement: Supplementary file 1 [file TID-24-73-s1.pdf]

Association and of MiR-27a-5p with chronic obstructive pulmonary disease and its role in human bronchial epithelial cell injury: a case-control and in vitro experimental study

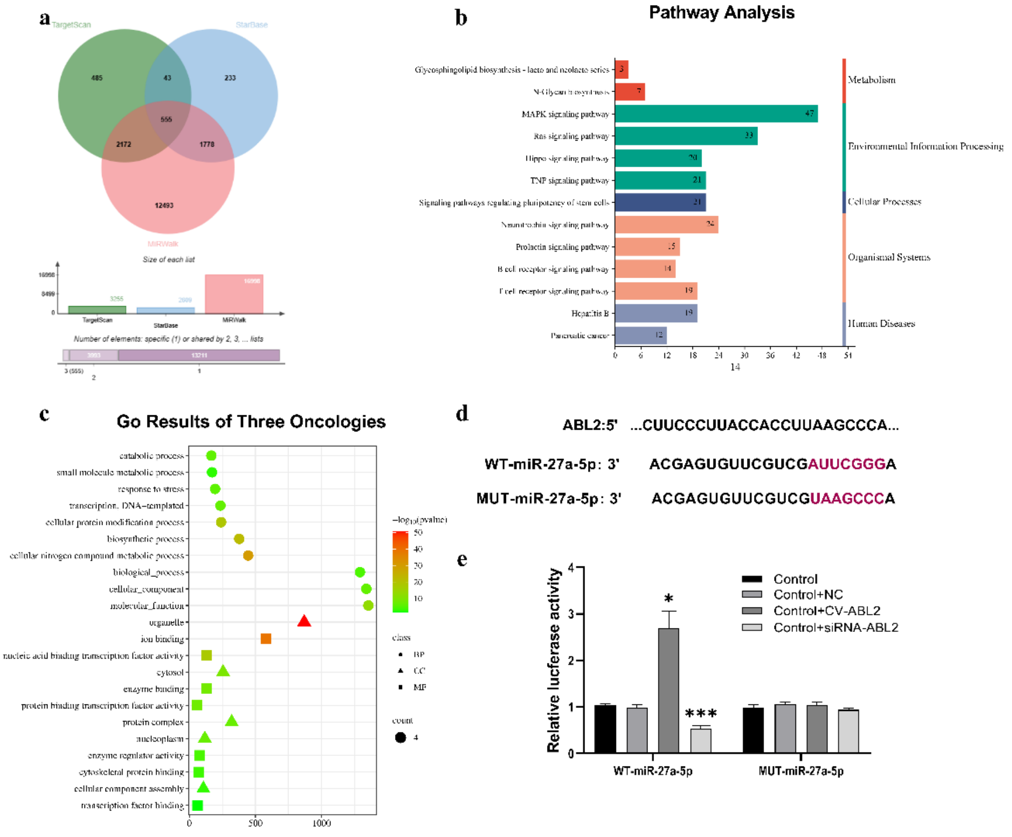

Experimental results of miR-27a-5p target gene prediction and verification in 2024. Analysis methods: Target gene intersection analysis, pathway enrichment analysis, GO functional annotation, dual luciferase reporter gene experiment; Sample source: Human bronchial epithelial cells

Figure S1. Bioinformatics prediction and validation of miR-27a-5p target genes

(a) Venn diagram and statistics of target genes predicted by TargetScan, miRDB, and miRTarBase databases, with the intersection representing common target genes. (b) Pathway analysis of common target genes. (c) GO functional annotation results. (d) Complementary binding sites between miR-27a-5p and the 3'UTR of ABL2. (e) Dual-luciferase reporter assay verifying the direct interaction between miR-27a-5p and ABL2. $P < 0.05$, $P < 0.001$

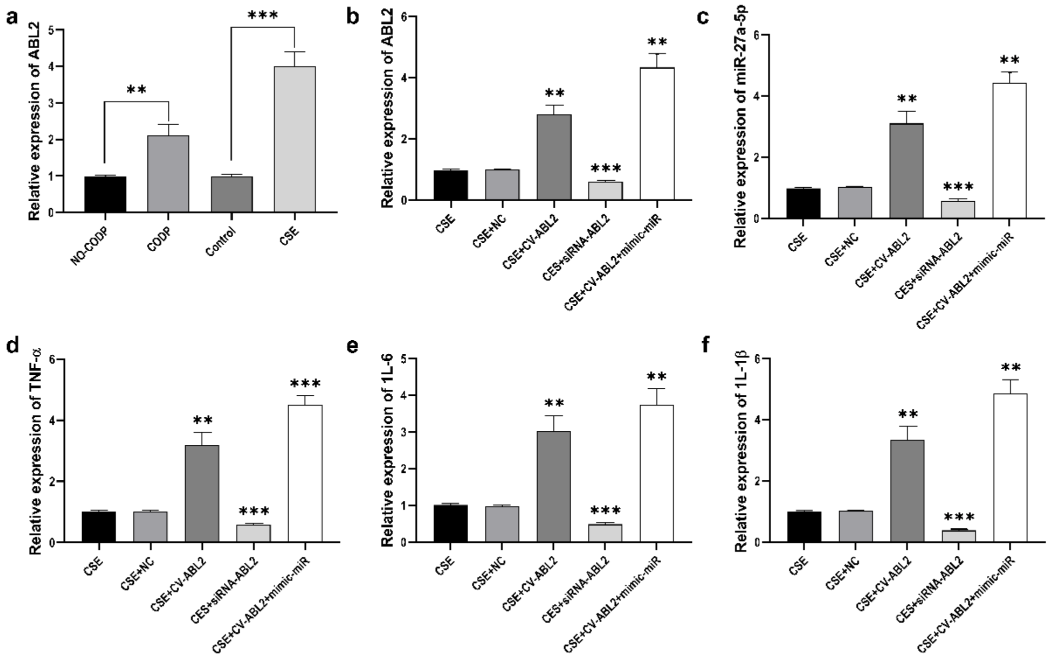

The regulatory effect of ABL2 on the expression of miR-27a-5p and inflammatory factors induced by CSE in in vitro experiments in 2024. Experimental groups: CSE group, CSE + NC group, CSE + ABL2 overexpression group, CSE + ABL2 silencing group, CSE + ABL2 overexpression + miR overexpression group; Sample size: 3 biological replicates for each group

Figure S2. Functional verification of the miR-27a-5p/ABL2 axis regulating the inflammatory response in CSE induced PBECs

(a) Relative expression of ABL2 in samples from NO-COPD and COPD patients, and in PBECs with or without CSE treatment. (b) ABL2 expression in CSE-treated PBECs after transfection with ABL2 overexpression vector, ABL2 siRNA, or negative control. (c) miR-27a-5p expression in CSE-treated PBECs under different ABL2 regulation conditions. (d-f) Relative expression of TNF-α (d), IL-6 (e), and IL-1β (f) in CSE-treated PBECs with various interventions targeting the miR-27a-5p/ABL2 axis. $P < 0.01$, $P < 0.001$

© 2026 Wang J. et al.
